# Clinical Characteristics of Coronary-to-Pulmonary Artery Fistula in Patients with Pulmonary Atresia and Ventricular Septal Defect

**DOI:** 10.3390/jcdd10010017

**Published:** 2023-01-03

**Authors:** Naofumi F. Sumitomo, Kazuki Kodo, Tadashi Inoue, Takayuki Oyanagi, Hiroyuki Yamagishi

**Affiliations:** Department of Pediatrics, Keio University School of Medicine, Tokyo 1608582, Japan

**Keywords:** coronary-to-pulmonary artery fistula, pulmonary atresia with ventricular septal defect, tetralogy of Fallot, major aortopulmonary collateral arteries

## Abstract

The existence of a coronary-to-pulmonary artery fistula (CPF) in pulmonary atresia with ventricular septal defect (PAVSD) potentially affects treatment; however, its clinical features have not been comprehensively described due to the disease’s rarity. We reviewed 69 cases from 42 studies to reveal the clinical overview of patients with CPF and PAVSD. Among the included patients, the male-to-female ratio was exactly 1:1, and only two patients (3%) exhibited the 22q11.2 microdeletion syndrome. Regarding anatomical features, CPFs originated from the left coronary artery in 65% of patients, and 62% had other major aortopulmonary collateral arteries. Thirty-nine percent of patients had a definitive CPF diagnosis at 0 years of age, whereas 10% were diagnosed in adulthood. Seventy percent underwent catheter angiography to obtain a definitive CPF diagnosis. Ninety-five percent of patients underwent cardiac surgery, and among them, 43% underwent palliative surgery, whereas 52% underwent one-stage repair. Four patients including three adult patients developed cardiac dysfunction due to myocardial ischemia, and three of them exhibited improved cardiac function after the intervention for CPF. Of all the patients, 88% survived and 12% died. The surgical strategy and prognosis were similar to those in PAVSD patients without CPF. This review provides detailed clinical phenotypes that are potentially useful in enhancing the management of patients with this rare disease.

## 1. Introduction

Coronary-to-pulmonary artery fistula (CPF) is a rare congenital anomaly with a prevalence rate ranging from 0.32% to 0.68% [1]. Most CPF cases are detected incidentally and display no clinical symptoms, whereas some cases present with significant hemodynamic effects, requiring surgical intervention [1]. While most CPF cases diagnosed in adulthood are solitary, CPF diagnosed in childhood is considered to be predominantly associated with congenital heart disease, especially pulmonary artery atresia with ventricular septal defect (PAVSD) [2]. Although some studies have provided brief summaries of the clinical features of CPF with PAVSD [2,3], the exact characteristics of this disease remain unknown due to the limited review of clinical information from a large patient population. This study aimed to summarize cases that have accumulated in previous reports to clarify the clinical overview of this rare disease.

## 2. Materials and Methods

### 2.1. Literature Search

A PubMed (https://www.nih.gov/) search was conducted in November 2021 using the following keywords: “coronary pulmonary fistula” and “tetralogy of Fallot (TOF)” or “pulmonary atresia.” Cardiac diseases such as pulmonary atresia with intact ventricular septum and TOF without pulmonary atresia were excluded. In total, 44 articles involving 77 patients were identified. Of these patients, eight were excluded due to inadequate data [4,5]. Finally, the remaining 69 patients, who were reported in 42 articles, were included in the present study [2,3,6,7,8,9,10,11,12,13,14,15,16,17,18,19,20,21,22,23,24,25,26,27,28,29,30,31,32,33,34,35,36,37,38,39,40,41,42,43,44,45]. Of these articles, 11 involving 21 patients (30%) were published before 1990 [9,10,11,12,13,14,15,16,17,18,19].

### 2.2. Data Collection

The following data were collected from the literature: sex, age at diagnosis, genetic abnormality, CPF’s anatomical origin, the area of pulmonary blood supply from CPF, other sources of pulmonary blood supply, type of aortic arch, the method of definitive CPF diagnosis, the presence of myocardial ischemia, history of treatment, and patient prognosis. In addition, in order to investigate whether the patients with pulmonary blood supply only originating from the coronary artery had clinical differences from other patients, we only extracted them and analyzed the above clinical characteristics in the same way.

In addition, we obtained the incidence rate of CPF in patients with PAVSD from previous studies, where available, and subsequently determined the same incidence rate in our hospital using medical records from 2000 to 2021 retrospectively for comparison with the reported data.

### 2.3. Statistical Analysis

Fisher’s exact test was used to compare the above-mentioned geometrical datasets based on the anatomical features of CPF and its area of pulmonary blood supply. Statistical analyses were performed using IBM SPSS Statistics, version 27 (IBM, Armonk, NY, USA). Statistical significance was set at *p* < 0.05.

## 3. Results

### 3.1. Patient Characteristics and Anatomical Features

Among the 46 patients, the male-to-female ratio was exactly 1:1. Of these patients, two out of 69 (3%) had the 22q11.2 microdeletion syndrome (22q11.2DS) [28,41]. According to a previous report, 1.3–10% of patients with PAVSD had concomitant CPF [3,6,7,8], and in our institution, one patient was diagnosed with CPF out of the 60 patients with PAVSD (1.7%) [45].

**Figure 1:**
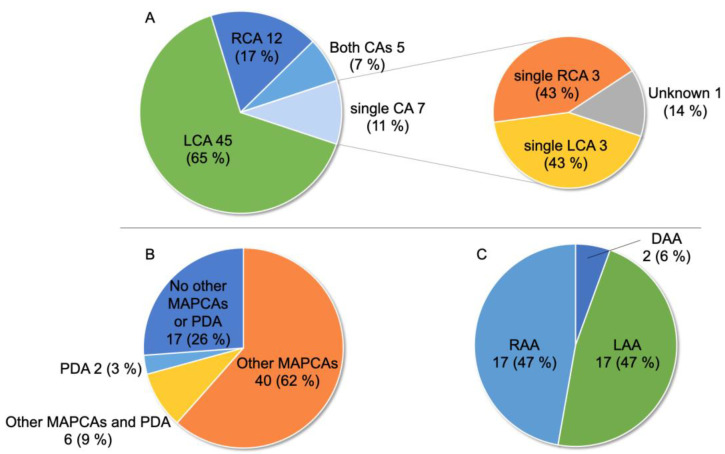
Anatomical features of coronary-to-pulmonary artery fistula (CPF) with pulmonary atresia with ventricular septal defect (PAVSD) in 69 patients. (**A**) Type of origin from the coronary cusp. (**B**) Type of blood supply to the lungs other than CPF (data not available in four patients). (**C**) Type of aortic arch (data not available in 33 patients). CA: coronary artery; DAA: double aortic arch; LAA: left aortic arch; LCA: left coronary artery; MAPCAs: major aortopulmonary collateral arteries; PDA: patent ductus arteriosus; RAA: right aortic arch; RCA: right coronary artery.

Figure 1 summarizes the anatomical features of CPF with PAVSD. Figure 1A describes the type of coronary cusp origin of CPF in all patients and revealed that 45 (65%) had CPF originating from the left coronary artery (LCA), whereas only 12 (17%), seven (11%), and five (7%) had CPF originating from the right coronary artery (RCA), single coronary artery, and both coronary arteries, respectively. Among the patients with CPF involving a single coronary artery, the number of coronary artery origins was similar between the left and right coronary cusps. Regarding pulmonary blood supply, 17 patients (26%) only had CPF as the source of the pulmonary flow of major aortopulmonary collateral arteries (MAPCAs), 40 (62%) had other MAPCAs in addition to CPF, six (9%) had both other MAPCAs and patent ductus arteriosus (PDA), two (3%) had both CPF and PDA, and the remaining four did not have detailed information (Figure 1B). Figure 1C shows the type of aortic arch in 36 patients. The number of patients with left and right aortic arches was the same (17 [47%]), and two patients had a double aortic arch. The frequency of the right aortic arch in these patients (43%) was approximately the same as that previously reported in patients with PAVSD [46].

Table 1 shows the relationship between the anatomical features and its area of pulmonary blood supply of the CPF. For 12 patients, the pulmonary blood flow distribution could not be assessed due to a lack of imaging data. Of the remaining 57 patients, the bilateral pulmonary blood supply originated from the CPF in 42 (74%), indicating the presence of a confluent pulmonary artery (PA) connected to CPF in these patients. In contrast, only six (10%) and nine (16%) patients had a left and right unilateral pulmonary blood supply originating from CPF, respectively. In patients who developed CPF from the LCA, 30 out of 35 (86%) patients received blood supply from CPF to both lungs, which was significantly higher than that in the other CPF groups (*p* = 0.026). In contrast, patients with CPF from the RCA had a significantly lower bilateral pulmonary perfusion incidence from CPF than the other CPF groups (*p* = 0.026).

### 3.2. CPF Diagnosis

**Figure 2:**
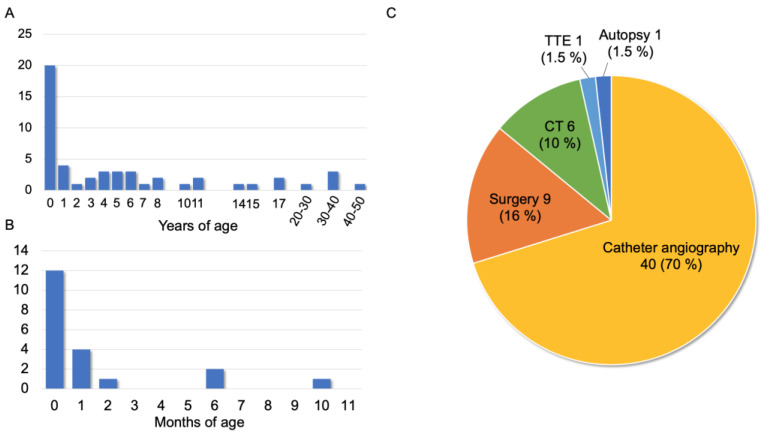
Diagnostic features of 69 patients with CPF and PAVSD. (**A**) Age distribution at the time of diagnosis of CPF (data not available in 18 patients). (**B**) Age at diagnosis for patients diagnosed under one year of age. (**C**) Definitive diagnosis method of CPF (data not available in 12 patients). CT: computed tomography; TTE: transthoracic echocardiography.

Figure 2A shows the age distribution of 51 patients at the time of CPF diagnosis. Twenty patients (39%) were diagnosed with CPF at 0 years of age, whereas five (10%) were diagnosed in their adulthood with symptoms of shortness of breath on exertion, chest pain, edema, and palpitation [17,27,30,34,40]. In addition, most CPF diagnoses under one year of age were made at 0 months of age (Figure 2B). Regarding definitive diagnosis based on the anatomical features of CPFs, 40 patients (70%) underwent catheter angiography, nine (16%) underwent intraoperative diagnosis, six (10%) underwent computed tomography (CT), and the remaining 12 cases had no information on diagnostic methods (Figure 2C). Only one patient was definitively diagnosed using echocardiography [3]. Fetal diagnosis of PAVSD was only mentioned in one case [41].

### 3.3. Cardiac Repair, Myocardial Ischemia, and Prognosis

**Figure 3:**
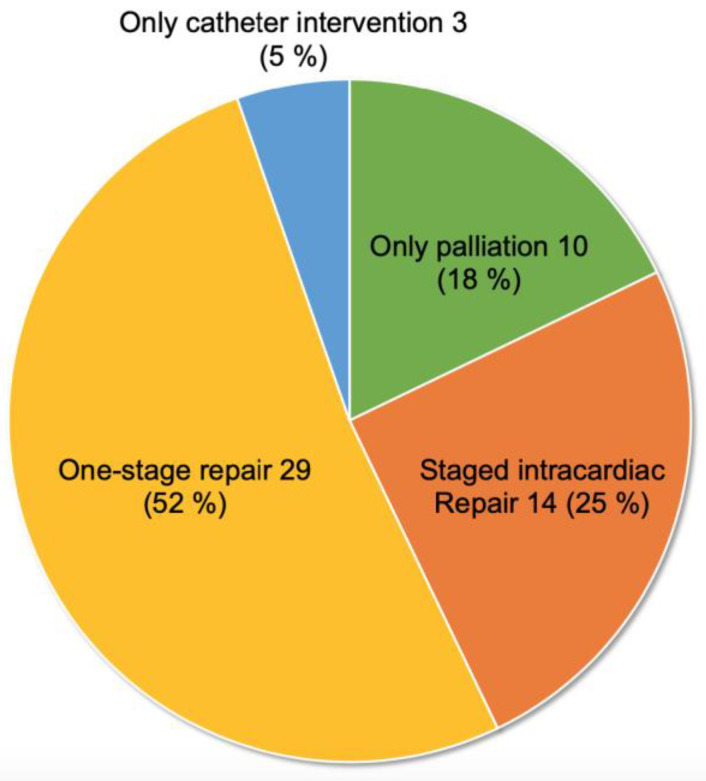
Treatment strategy of 69 patients with CPF and PAVSD (data not available in 13 patients).

Of 56 patients with information about treatment, 53 (95%) underwent cardiac surgery (Figure 3). Among them, 29 (52%) underwent one-stage repair. However, 24 (43%) underwent palliation upon initial surgery, and more than half of them (n = 14) subsequently underwent intracardiac repair. Almost all of the CPF was divided, ligated, or clipped at the nearside of the coronary artery during the palliative surgery or intracardiac repair. The procedure of palliation included any systemic to pulmonary shunt, unifocalization, or ligation of MAPCAs, and palliative reconstruction of the right ventricular outflow tract. Most intracardiac repair was the usual procedure with the closure of ventricular septal defect and the reconstruction of the right ventricular outflow tract. Three patients underwent only catheter intervention during adulthood [27,30,34]. These interventions were described as a stent placement for CPF to increase the pulmonary blood flow in a patient with deep cyanosis [27], a device occlusion of CPF to improve congestive heart failure [30], and a coil occlusion of CPF for patients with myocardial ischemia [34].

**Figure 4:**
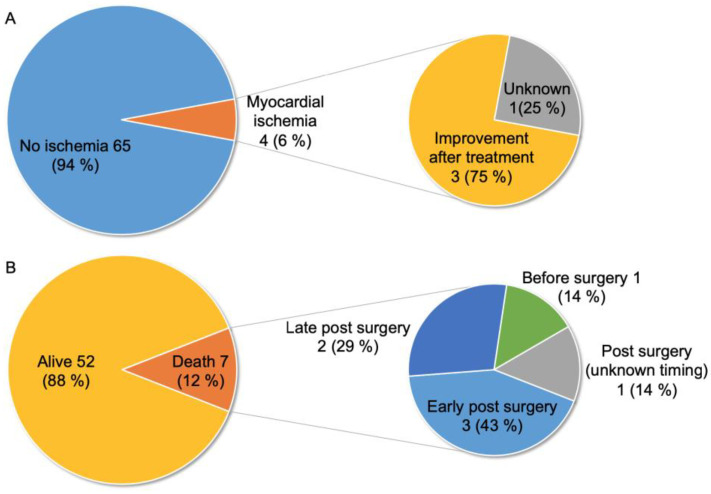
Perioperative status and prognosis of 69 patients with CPF and PAVSD. (**A**) Presence of myocardial ischemia and post-treatment changes. (**B**) Patient prognosis and mortality characteristics (data not available in 10 patients).

Figure 4 shows the patients’ perioperative statuses. Four patients (6%) had cardiac dysfunction due to myocardial ischemia, three of whom exhibited improved cardiac function after intervention for CPF (Figure 4A) [30,31,34,38]. Of 59 patients, 52 (88%) survived, and seven (12%) died [7,8,9,12,18,19,24], five of which were reported before 2000. One and six patients died preoperatively and postoperatively, respectively, including three early deaths (within 1 month after surgery) and two late deaths after surgery (Figure 4B). The cause of death included heart failure in one preoperative and one early postoperative patient [7,18], acute renal failure in one early postoperative patient [19], sudden death in one late postoperative patient [24], and unknown in the remaining three.

### 3.4. Comparison of Clinical Features in Patients with Pulmonary Blood Supply Only Originating from the Coronary Artery and Other Patients

**Figure 5:**
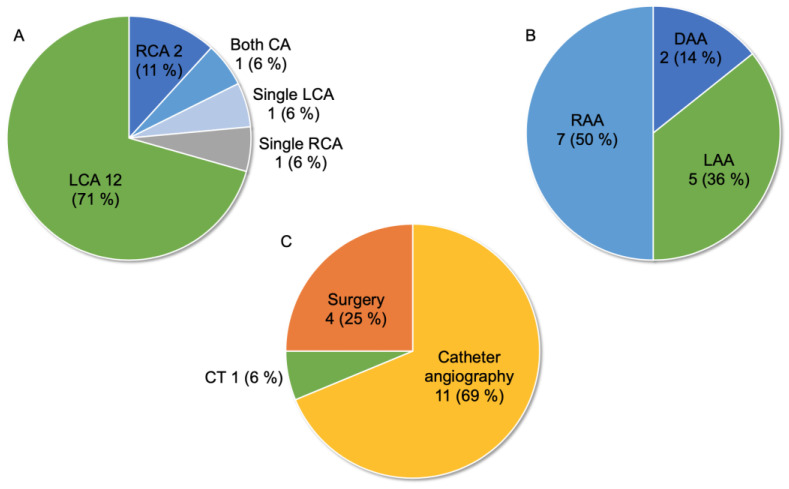
Clinical features of 17 patients with CPF and PAVSD who had pulmonary blood supply only originating from the coronary artery. (**A**) Type of origin from the coronary cusp. (**B**) Type of aortic arch (data not available in three patients). (**C**) Definitive diagnosis method of CPF (data not available in one patient). CA: coronary artery; CT: computed tomography; DAA: double aortic arch; LAA: left aortic arch; LCA: left coronary artery; RAA: right aortic arch; RCA: right coronary artery.

Figure 5 shows the clinical features of 17 patients with CPF and PAVSD who had pulmonary blood supply only originating from the coronary artery. Regarding the type of coronary cusp origin of CPF, 12 (71%) had CPF originating from the LCA, whereas two (11%), one (6%), one (6%), and one (6%) had CPF originating from the RCA, both coronary artery, single LCA, and single RCA, respectively (Figure 5A). Figure 5B shows the type of aortic arch in 14 patients. The number of patients with left and right aortic arches was 5 (36%) and 7 (50%), respectively, and two patients had a double aortic arch. Definitive diagnosis based on CPF’s anatomical features was summarized in Figure 5C, and 11 patients (69%) underwent catheter angiography, four (25%) underwent intraoperative diagnosis, one (6%) underwent CT, and the remaining one was unknown. These clinical features showed almost the same tendency compared to all patients.

**Figure 6:**
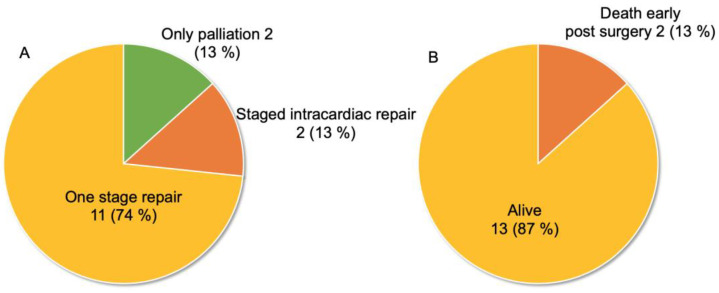
Treatment strategy and prognosis of 17 patients with CPF and PAVSD who had pulmonary blood supply only originating from the coronary artery. (**A**) Treatment strategy (data not available in two patients). (**B**) Patient prognosis and mortality characteristics (data not available in two patients).

Table 2 shows the relationship between the anatomical features and its area of pulmonary blood supply in the CPFs of 52 patients except for the 17 with pulmonary blood supply only originating from the coronary artery (data not available in 12 patients). There was no significant difference in the number of patients in each group, however, the tendency of the number of patients in each group was considered to be similar to that of all patients. Figure 6 shows the treatment strategy and prognosis of 17 patients with CPF and PAVSD who had pulmonary blood supply only originating from the coronary artery (data not available in two patients). Although the rate of one-stage repair was higher at 74% in this group than that of all 69 patients (Figure 6A)—presumably due to the anatomical simplicity of the pulmonary vessels in this group—the survival rate was almost the same at 87% (Figure 6B).

## 4. Discussion

### 4.1. Basic Characteristics of the Patients

In the present study, the number of male and female patients was equal among patients with CPF and PAVSD, a phenomenon that also emerged among those with TOF [47]. The prevalence of CPF in patients with PAVSD tended to exceed that in patients without congenital heart disease (1.3–10% vs. 0.32–0.68%) [1]; however, substantial variability was noted in the frequency of CPF in patients with PAVSD across existing studies including ours, possibly because many of these studies reported CPF frequencies based on a relatively small number of single-center cases. Other reasons could be regional and racial differences in the frequency of CPF; nonetheless, highlighting these factors based exclusively on this study is difficult due to the lack of data. To clarify this issue, examining a large number of cases from more institutions is warranted in future studies.

### 4.2. Anatomical, Embryologic and Genetic Features

Historically, Hackensellner proposed the involution–persistence hypothesis to explain the embryologic mechanism of CPF. Originally, six coronary artery anlagen appeared in the base of the fetal aortopulmonary truncus: three on the aortic side and three on the pulmonary side. Under normal conditions, the coronary arteries arise from two persistent anlagen in two aortic sinuses of Valsalva, while the anlagen in the third aortic sinus and in all three pulmonary sinuses either undergo rapid involution or are not formed. Therefore, the anomalous origin of one or both coronary arteries from the pulmonary artery potentially results from the combination of the abnormal persistence of one or more of the normally involuted pulmonary artery coronary anlagen and abnormal involution of one or more of the normally persistent aortic coronary anlagen [48]. With regard to the formation of the coronary artery base, recent animal studies have suggested that the coronary vascular plexus formed around the aortopulmonary–truncus base invades the two aortic sinuses of Valsalva from the adventitia side, causing aortic-wall apoptosis, to form multichannels. Thereafter, vascular smooth muscle cells are mobilized to the immature multichannel coronary vessel wall connected to the aorta and facilitate vessel remodeling to form mature connections of the RCA and LCA [49]. Molecularly, vascular endothelial growth factor C stimulates vessel growth around the aortopulmonary truncus, and the expression of islet-1, a transcription factor essential for cardiac development, allows cardiomyocytes to differentiate specifically in the aortic wall, where they support vessel growth and facilitate peritruncal–vessel connections to the aorta’s luminal endothelium [50]. Failure to complete either step potentially results in abnormal coronary-artery patterning including CPF.

Although our results indicate that most patients had CPF originating from the LCA, exhibiting consistency with findings from previous reports [2,3], the reason CPF tends to form with the LCA rather than with other coronary-artery patterns remains obscure. Based on the mechanism underlying the development of the normal coronary arterial orifices and CPF described above, we hypothesize that the LCA’s closer anatomical proximity to the main pulmonary trunk than that of the RCA may contribute to its susceptibility to fistula formation in the anlagen between the LCA and PA. In addition, our study revealed that CPF originating from the LCA tends to be distributed toward the bilateral PA more than toward other originating patterns. This may be because CPF, connecting from the LCA to the main PA, maintains the blood flow supply of the main/central PA effectively and promotes central PA development during the fetal period, resulting in the confluency of the bilateral PA.

Intriguingly, patients with CPF and PAVSD had a significantly low incidence of 22q11.2DS (3%), whereas the same microdeletion was more frequently detected in 14–53% of PAVSD patients without CPF [51]. Because this study included articles published in an era when 22q11.2DS had not yet been diagnosed, the incidence described above may be underestimated. However, upon exclusively considering recent reports published after 2000, the association of 22q11.2DS was found in only two out of 42 patients, yielding a persistently low incidence of 4.7%. In patients with 22q11.2DS, the central PA is typically hypoplastic or absent due to hypoplasia of the sixth pharyngeal arch artery during fetal development [52,53,54]. Accordingly, we hypothesize that anlagen of the coronary artery on the PA side may also be too hypoplastic to form a fistula with the coronary artery in patients with 22q11.2DS.

### 4.3. Diagnostic Methods

Our results demonstrate that several patients were diagnosed during early infancy when they were small. In such cases, the accurate identification of small coronary vessels originating from a large CPF using echocardiography or CT is generally challenging; thus, invasive catheter angiography is required to confirm the precise anatomical feature in most cases. In contrast, definitive CT diagnoses have usually been made in patients with a large physique, whereas only limited cases of CT diagnosis in infancy have been reported [38,45].

Although most CPF cases were diagnosed preoperatively, others were not, even after 2000, and they were incidentally confirmed during surgery [8,26,37]. In several CPF cases, echocardiography typically revealed significant dilation of the coronary artery ostium, thus posing as a clue to the existence of CPF [2,3,31,35,36,41,42]. Therefore, careful screening of the structure of the proximal coronary artery should be performed in PAVSD to enable CPF identification. If CPF existence is suspected, the precise anatomical diagnosis of small vessels should be made using angiography or CT to formulate an accurate surgical plan.

### 4.4. Treatment and Prognosis

In patients with PAVSD and MAPCA, the rate of one-stage repair has been reported to be 51–93% [49]. In the present study, approximately half of the operated patients underwent one-stage repair, which was not significantly different from that of patients without CPF. The CPF needs to be divided or ligated during surgery, and presumably, the procedure can be performed simultaneously with both palliative surgery and intracardiac repair. Thus, the presence of CPF does not appear to significantly affect the entire surgical strategy for patients with PAVSD. In patients with PAVSD and MAPCA, the early and late mortality rates have been reported to be 0–11% and 0–16%, respectively [49]. The present study demonstrated that CPF with PAVSD had a total mortality rate of 12%, almost equivalent to that in past reports. Therefore, CPF is considered not to alter the prognosis of patients with PAVSD significantly if appropriate treatment for CPF is administered.

### 4.5. Myocardial Ischemia

As previously reported, CPF was rarely associated with myocardial ischemia in the present study [2,3]. Theoretically, CPF potentially causes this type of ischemia due to coronary steal to the lungs, which have low vascular resistance; however, this phenomenon is considered rare. In fact, an enlarged coronary orifice maintains coronary perfusion as a compensatory mechanism [3]. In addition, another possible reason for maintaining coronary perfusion in patients with CPF is that PA pressure is more likely to maintain coronary diastolic pressure than the coronary artery fistula to the atrium or ventricle, which is perceived to have lower coronary artery diastolic pressure than CPF due to low ventricular filling pressure. Of all four patients with myocardial ischemia in the present study, three were adolescents or older and were either unoperated or exclusively underwent palliative surgery [30,31,34]. The remaining patient was an early infant associated with severe aortic valve regurgitation that was considered to promote coronary steal along with CPF [38]. In addition, all patients presented with myocardial ischemia before intracardiac repair, implying that they had cyanosis upon ischemia diagnosis. Hence, although the incidence of myocardial ischemia in CPF is low, long-term, unrepaired patients with chronic cyanosis or other coronary steal mechanisms may be aware of myocardial ischemia.

### 4.6. Limitations and Future Directions

Because the data in this study depended on several previous case reports, the unavailability of some patient information was inevitable. Second, since the collected literature included articles published before 1990, the diagnostic methods or prognoses reported therein might have varied from the current ones. Moreover, the current study included patients with pulmonary blood supply only originating from the coronary artery, however, it is argued whether these patients and others with MAPCAs have exactly the same nature of the pulmonary vessel. Although many of the clinical characteristics were similar between these two groups of patients, further investigation is required to delineate their histological and embryological nature of pulmonary vascular. Finally, the number of patients investigated in this study was relatively small due to the rarity of the disease. Therefore, ongoing investigations should be conducted as more cases accumulate to obtain further details regarding the patient clinical features.

## 5. Conclusions

This study was the first to provide an exhaustive clinical description of PAVSD patients with CPF. Our findings suggest that although PAVSD patients with CPF have certain unique clinical characteristics that deviate from those without CPF, their surgical outcomes are similar to those of individuals without CPF; furthermore, the incidence of myocardial ischemia was found to be low in this patient population.

## Figures and Tables

**Table 1:** Anatomical type of CPF and its area of pulmonary blood supply in 69 patients (data not available in 12 patients).

	Left Lung	Right Lung	Bilateral Lung	Total
LCA	1 ^†^	4	30 *	35
RCA	4 *	2	5 ^†^	11
Both CA	0	1	3	4
Single CA	1	2	4	7
Total	6 (10%)	9 (16%)	42 (74%)	57

CA: coronary artery; LCA: left coronary artery; RCA: right coronary artery. * indicates that the number is significantly higher than the others (*p* < 0.05). ^†^ indicates that the number is significantly lower than the others (*p* < 0.05).

**Table 2:** Anatomical type of CPF and its area of pulmonary blood supply in 52 patients except for the group with pulmonary blood supply only originating from the coronary artery (data not available in 12 patients).

	Left Lung	Right Lung	Bilateral Lung	Total
LCA	1	4	18	23
RCA	4	2	3	9
Both CA	0	1	2	3
Single CA	1	2	2	5
Total	6 (1%%)	9 (12%)	25 (63%)	40

CA: coronary artery; LCA: left coronary artery; RCA: right coronary artery.

## Data Availability

Not applicable.
